# SQUID-based detection of ultra-low-field multinuclear NMR of substances hyperpolarized using signal amplification by reversible exchange

**DOI:** 10.1038/s41598-017-13757-7

**Published:** 2017-10-18

**Authors:** K. Buckenmaier, M. Rudolph, C. Back, T. Misztal, U. Bommerich, P. Fehling, D. Koelle, R. Kleiner, H. A. Mayer, K. Scheffler, J. Bernarding, M. Plaumann

**Affiliations:** 1 High-Field Magnetic Resonance Center, Max Planck Institute for Biological Cybernetics, Spemannstr. 41, 72076 Tübingen, Germany; 2 Physikalisches Institut and Center for Quantum Science (CQ) in LISA+, University of Tübingen, Tübingen, Germany; 3 Institute of Inorganic Chemistry, University of Tübingen, Tübingen, Germany; 4 Department for Biometrics and Medical Informatics, Otto-von-Guericke University, Magdeburg, Germany

## Abstract

Ultra-low-field (ULF) nuclear magnetic resonance (NMR) is a promising spectroscopy method allowing for, e.g., the simultaneous detection of multiple nuclei. To overcome the low signal-to-noise ratio that usually hampers a wider application, we present here an alternative approach to ULF NMR, which makes use of the hyperpolarizing technique signal amplification by reversible exchange (SABRE). In contrast to standard parahydrogen hyperpolarization, SABRE can continuously hyperpolarize 1 H as well as other MR-active nuclei. For simultaneous measurements of 1 H and 19 F under SABRE conditions a superconducting quantum interference device (SQUID)-based NMR detection unit was adapted. We successfully hyperpolarized fluorinated pyridine derivatives with an up to 2000-fold signal enhancement in 19 F. The detected signals may be explained by two alternative reaction mechanisms. SABRE combined with simultaneous SQUID-based broadband multinuclear detection may enable the quantitative analysis of multinuclear processes.

## Introduction

Signal Amplification By Reversible Exchange (SABRE) is a relatively new technique to produce continuous hyperpolarization to boost nuclear magnetic resonance (NMR) signals and is even applied for magnetic resonance imaging (MRI)^1,2^. SABRE is based on a symmetry-breaking mechanism that converts the parahydrogen (para-H_2_) spin order into a non-Boltzmann polarization^1,3,4^. High-field NMR measurements show signal enhancements of up to a factor of 10^5^ for ^1^H. The underlying mechanism is based on the interaction of para-H_2_ with a substrate of interest via an Ir-based catalyst system^1^.

Gong *et al*. first reported the use of SABRE at low-field in 2010^5^. Further low-field (10–500 mT) examinations for the standard para-H_2_ induced polarization (PHIP) approach, where hydrogen atoms were added to a double or triple bond^6,7^, were done by Hamans *et al*.^8^ and Theis *et al*.^9,10^. In general, the advantage of SABRE against standard-PHIP is the possibility that the enhanced substrates can be continuously re-hyperpolarized by supplying a steady flow of parahydrogen^11,12^. It is important to note that most heteronuclei require special pulse sequences, or one has to lower the field strength to perform an efficient polarization transfer. Thus, for high-field MR, dedicated solutions have been developed such as SABRE-SHEATH^13^ or mechanically challenging shuttle mechanisms^14^. Most of the experiments have focused on the detection of hyperpolarized molecules in high magnetic fields, on increasing the number of hyperpolarized substrates^15,16^, or on measuring extracts of biofluids where spin densities down to sub-μM concentrations could have been detected^17^. The mechanism relies on transforming the spin order of the para-H_2_ singlet state into nuclear spin polarization of Ir hydride protons when an exchange reaction is performed in a low magnetic field^1,18,19^. The required field strength (2–10 mT) of the polarization transfer reaction to further spin-½ nuclei of the substrate such as pyridine is strongly dependent on the coupling constants^1,20,21^. So far the theoretical background of SABRE is based on level anti-crossings between magnetic field independent J-coupling energy levels and the magnetic field dependent chemical shift^11,22,23^.

Low-field and ultra-low-field (ULF, <10 mT) NMR provide an optimal and in comparison to high-field NMR less costly tool for investigating the polarization transfer mechanism. Even MR imaging can be realized at ultra-low fields^24–32^. More important, certain experiments that require complex and expensive technical efforts at high fields can be easily realized at ultra-low fields, such as the simultaneous detection of several nuclei^33^, the separation of detection field and polarizing field, or the variation of the polarizing field. However, a wider use of ULF NMR is still hampered by the inherent low signal-to-noise ratio (SNR), as the thermal equilibrium magnetization is increasing with the polarizing field, which is typically in the range of several mT as compared to high-field NMR with up to 23.5 T. Additionally, ULF NMR signals are in the range of some kHz (about 2 kHz at the earth’s magnetic field), which renders the signal in detection coils too low.

A combined strategy provides a solution to increase the SNR dramatically: first, detection is realized by a superconducting interference device (SQUID) that is very sensitive and can detect magnetic field down to some fT; second, the signal is enhanced by using the hyperpolarization technique SABRE.

ULF NMR provides the advantage that the polarizing field *B*_*p*_ > *B*_0_, where *B*_0_ is the detection field of the NMR signal, can be easily and reproducibly adapted to the individual field strengths required for the optimum polarization transfer of specific substances^34^. The *B*_*p*_ field is usually realized by electromagnetic coils which allow field changes within milliseconds. Also imaging in the vicinity of metals is possible without distortion artefacts^35^. The sensitivity of different magnetic field detectors used for ULF MRI^36,37^, such as for example atomic magnetometers, is comparable to the already widely used SQUIDs. A SQUID-based system has not only a higher signal to noise ratio (SNR) compared to a system using a Faraday coil at ULF^38^, but also can detect the NMR signal of multiple nuclei simultaneously. SQUIDs are broadband detectors, which are able to detect the magnetic flux directly, rather than the change of the magnetic field, making them sensitive from DC up to the GHz range^39^.

Another advantage is that the NMR signal can be detected via a second order superconducting gradiometer, which acts as a surface coil^39^. The open geometry and the small field strengths needed for detecting the NMR signal makes a SQUID based system combinable with imaging techniques such as magnetoencephalography (MEG)^40,41^, where SQUIDs can be used to detect the MEG and NMR signals.

**Figure 1:**
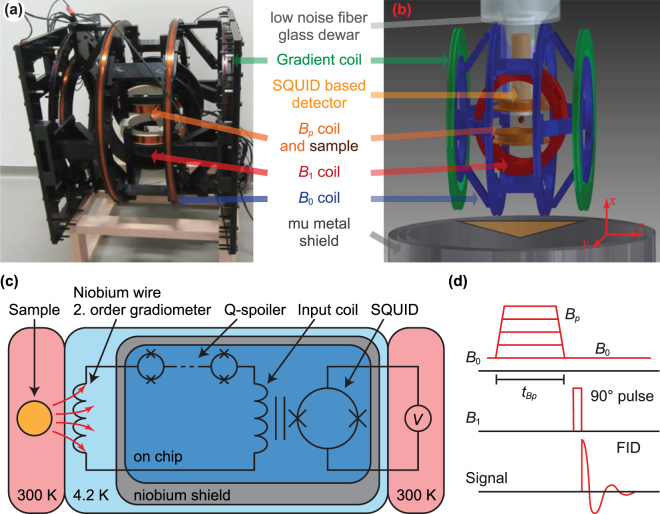
Photo (**a**), scheme of the ULF MRI system (**b**), scheme of the SQUID based magnetic field detector (**c**) and the pulse sequence used for the ULF NMR measurements (**d**).

The design of our home-built ULF MRI system is derived from previously described detection architectures^33,41,42^. It consists of a tetracoil^43^ with radius 260 mm for generating the *B*_0_ field along the *z* axis, a *B*_1_ coil in a Helmholtz configuration with radius 145 mm oriented perpendicular to *B*_0_ in the *y* direction, a prepolarizing *B*_*p*_ Helmholtz coil with radius of 90 mm oriented along the *x* axis, and a gradient coil in a Maxwell configuration with radius 306 mm for shimming along the *z* axis. All coils are driven by commercially available current amplifiers (Hubert A110-16-QE, Kepco BOP 100-4 ML, Highfinesse BCS 3/12). The DC current sources are heavily filtered with pi-filters. The amplifiers for the *B*_1_ and *B*_*p*_ coil were galvanically separated from the whole setup during the readout of the signal via mechanical relays. The heart of the system, the SQUID-based magnetic field detector, is sitting inside a liquid- helium filled low-noise fiber glass Dewar [see Fig. 1(a) and (b)]^44,45^. The SQUID itself sits inside a Niobium shield preventing background noise to couple directly into the SQUID [see Fig. 1(c)]. A pickup coil in a second-order gradiometer configuration, with a loop diameter of 40 mm and a baseline of 40 mm, is used to couple the NMR signal to the SQUID via an input coil. The gradiometer is sensitive to the sample, which locates 12 mm (the hot-to-cold distance of the Dewar) below the lowest loop, and it rejects signals from distant noise sources, because such sources usually exhibit only a small gradient. In series with the gradiometer and input coil, a current-limiting array of SQUIDs (Q-spoiler) with a critical current *I*_*c*_ acts as a superconducting short, as long as the induced current in the gradiometer is less than *I*_*c*_. Above *I*_*c*_ the junctions become dissipative, limiting the maximum current induced in the gradiometer and protecting the SQUID. Large currents are, for example, induced by the pulsed magnetic fields needed in ULF MRI.

Since SQUIDs are sensitive to signals from DC up to the GHz range, the whole system is placed inside a cylindrical mu-metal shield with a diameter of 780 mm for magnetic shielding and inside a cubic steel-shielding chamber with an edge length of 2.8 m for shielding RF noise.

To allow for continuous hyperpolarization, a cylindrical/spherical PEEK vessel with 2 ml volume serving as reaction chamber was adapted. The para-H_2_ was continuously delivered at the bottom of the vessel and bubbled continuously through the sample. At the top part, a small hole permitted the para-H_2_ outflow. Since the evaporation rate of the substrate dramatically increases with the flow rate of the para-H_2_ and capillary action in combination with overpressure due to the para-H_2_ removes liquid from the vessel, a reservoir behind the hole in the top part was installed together with a backflow tube. The backflow tube feeds the liquid from the reservoir back to the bottom of the reaction chamber. A typical measurement session required about 2 to 3 hours. At a para-H_2_ flow rate of approx. 1.5 l/h, roughly 3–4 ml of the substrate evaporated. By filling the reservoir with additional 5 ml we were able to measure during the whole session without refilling. The temperature of the sample stayed constantly at room temperature during the whole measurement. The para-H_2_ was produced on-site during the experiment with a home-made para-H_2_ generator operating at about 21 K–30 K. The design of the generator is based on ref. ^46^. The concentration of para-H_2_ (in comparison to orthohydrogen) was determined before the experiments by using a home-made temperature-stabilized thermal-conductivity cell based on a heated tungsten filament^47–50^. With this method a quantitative analysis of the para-H_2_ concentration is difficult, however qualitatively we could see that the concentration was much higher than the equilibrium concentration at liquid nitrogen temperature (77 K).

A simple free-induction-decay (FID) pulse sequence served for acquiring the NMR signal and is shown in Fig. 1(d). A prepolarizing pulse with variable amplitude *B*_*p*_ and variable length *t*_*Bp*_ was used to increase the *B*_0_ field strength. After the adiabatic switch-off of the *B*_*p*_ field the sample magnetization is aligned with *B*_0_ and a 90° *B*_1_ pulse followed. A double resonant pulse, for example, a *B*_1_ pulse exhibiting in its Fourier transform two peaks at the Larmor frequencies of nucleus X and Y, can be used to excite the magnetization of multiple nuclei simultaneously. In this study we used single resonant pulses with one exception in the SI (see spectrum in Figure S1). Subsequently, the FID was read out.

As model compounds 3,5-bis(trifluoromethyl)pyridine, ethyl-5-fluoronicotinic acid and 3-fluoropyridine were chosen to compare the hyperpolarizability of ^1^H and ^19^F quantitatively under ULF conditions. All measurements were performed in presence of an Ir catalyst [Ir(COD)(IMes)(Cl)]^51^. Substrates as well as the catalyst were dissolved in methanol and injected at room temperature into the reaction chamber before starting the first measurement. The exact mixture of the three substances was 7 mg of [Ir(COD)(IMes)(Cl)] and 0.23 mmol of the fluorinated pyridine derivative, dissolved in 10 ml methanol. The samples were not degassed.

The detection field *B*_0_ was ~150 µT (about three times stronger than the earth’s magnetic field). Accordingly, the Larmor frequencies of the hyperpolarized nuclei were ~6140 Hz for ^1^H and ~5770 Hz for ^19^F (see peak positions in SI Figure S1). The field strength of ~150 µT was chosen, because the noise spectrum around 6 kHz did not show any additional noise due to external noise sources. Test experiments on solutions of the compounds and catalyst in methanol but without para-H_2_ show small MR signals of the methanol, which increases linearly with *B*_*p*_. Since this signal is much smaller than the hyperpolarized signal of the substrate, no deuterated methanol was used.

**Figure 2:**
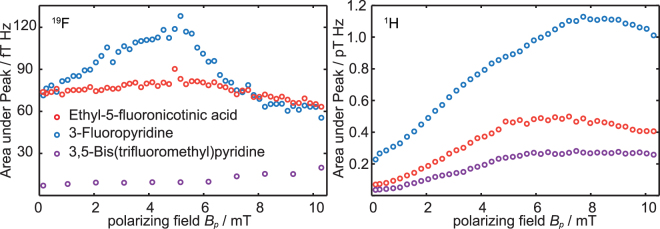
Integrated peak signals vs. *B*_*p*_ amplitude for the ^19^F (left) and ^1^H (right) signals of all three substances.

To determine the influence of the polarizing field *B*_*p*_ on the achievable signal enhancements experiments were performed using the sequence shown in Fig. 1d. The *B*_*p*_ field strength was varied between 0.144 mT (the *B*_0_ field) and 10.3 mT. The acquired signal was Fourier transformed, and the absolute value of the signal was integrated over the range of the multiple peak structure (≈20 Hz around the excitation frequency). The results show (Fig. 2) that the signal enhancement is not only influenced by the polarizing field, but also by the substance to be hyperpolarized and the corresponding nuclei. Interestingly, the ^19^F signals of the monofluorinated compounds exhibit only a weak dependence on the polarizing field when compared to the according ^1^H signals. The field strengths that yield maximum signal enhancement for the ^19^F and the ^1^H signal of the substances were also different. The *B*_*p*_ field for the maximum ^19^F signal enhancement is lower for the two monofluorinated pyridine derivatives lower than for ^1^H. 3,5-Bis(trifluoromethyl)pyridine seems to exhibit a different behavior. Here, the *B*_*p*_ field dependency of the ^19^F is even less pronounced than for all the other substrates. The small increase of the area-under-peak for stronger *B*_*p*_ field strengths is due to an increase in noise, which becomes dominant here, because of the small SNR.

**Figure 3:**
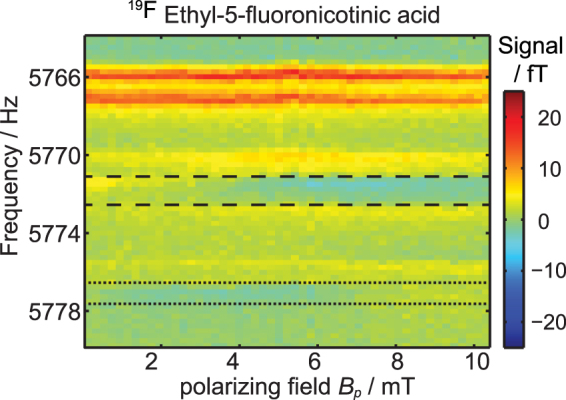
^19^F spectra of ethyl-5-fluoronicotinic acid as a function of *B*_*p*_. The signal intensity is given in fT. For *B*_*p*_ < 1 mT all visible NMR peaks are positive. With increasing *B*_*p*_ the peaks between, respectively, the dashed and the dotted lines become negative.

A more detailed investigation can be made by plotting the spectra (real part of the MR signal) as a function of the *B*_*p*_ field. Due to J-coupling, multiple peaks can be observed. For 3-fluoropyridine and 3,5-bis(trifluormethyl)pyridine the signal amplitudes of all resonances are correlated. The same could be observed for the ^1^H signal of ethyl-5-fluoronicotinic acid. An exception is observed for the ^19^F signal of ethyl-5-fluoronicotinic acid (see Fig. 3): the peak intensity below 5771 Hz and between 5773 Hz and 5776 Hz seems to be correlated. However, the peak at 5772 Hz (between dashed lines) and at 5778 Hz (between dotted lines) is changing its phase for increasing *B*_*p*_. The sequence parameters are listed in Table 1, measurement number 1 to 6.

**Table 1:** Overview of the sequence parameters used for all presented ultra-low-field measurements.

**#**	**Substance**	**Avgs**.	**TR[s]**	***B***_***p***_ **time[s]**	***B***_***p***_ **field[mT]**	***B***_**1**_ **Freq**.**[Hz]**	**Nucleus**
1	Ethyl-5-fluoronicotinic acid	5	6.5	2	0.144–10.3	5775	^19^F
2	3-Fluoropyridine	5	6.5	2	0.144–10.3	5775	^19^F
3	3,5-Bis(trifluoromethyl)pyridine	50	3.5	2	0.144–10.3	5775	^19^F
4	Ethyl-5-fluoronicotinic acid	5	8.5	4	0.144–10.3	6140	^1^H
5	3-Fluoropyridine	5	8.5	4	0.144–10.3	6140	^1^H
6	3,5-Bis(trifluoromethyl)pyridine	5	8.5	4	0.144–10.3	6140	^1^H
7	Ethyl-5-fluoronicotinic acid	10	1.6–10.6	0–10	3.1	5775	^19^F
8	3-Fluoropyridine	10	1.6–10.6	0–10	5.2	5775	^19^F
9	3,5-Bis(trifluoromethyl)pyridine	25	1.5–10.5	1.5–10.5	0.14	5775	^19^F
10	Ethyl-5-fluoronicotinic acid	10	1.6–10.6	0–10	6.2	6140	^1^H
11	3-Fluoropyridine	10	1.6–10.6	0–10	7.7	6140	^1^H
12	3,5-Bis(trifluoromethyl)pyridine	10	1.6–10.6	0–10	7.7	6140	^1^H
13	Ethyl-5-fluoronicotinic acid	50	9.5	9.5	0.144	5775	^19^F
14	3-Fluoropyridine	50	9.5	4	5.2	5775	^19^F
15	3,5-Bis(trifluoromethyl)pyridine	200	5.25	5.25	0.144	5775	^19^F
16	Ethyl-5-fluoronicotinic acid	50	9.5	4	6.2	6140	^1^H
17	3-Fluoropyridine	50	9.5	4	7.7	6140	^1^H
18	3,5-Bis(trifluoromethyl)pyridine	100	10.1	5	7.7	6140	^1^H

The measurements support previous results that the polarization transfer from ^1^H to further spin-1/2 nuclei is field-dependent even at ultra-low fields^52,53^. In the next set of experiments we investigated the signal enhancement of ^1^H and ^19^F and the magnetization transfer between both nuclei when applying the SABRE technique. It was observed that a strong magnetization transfer reaction to ^1^H and ^19^F nuclei takes place in the range of 0–10 mT. However, while the transfer to ^1^H nuclei of pyridine derivatives was strongly field-dependent, the field dependence for ^19^F hyperpolarization is much less pronounced (see Fig. 2). To the best of our knowledge, this is the first time that such behavior could be detected.

**Figure 4:**
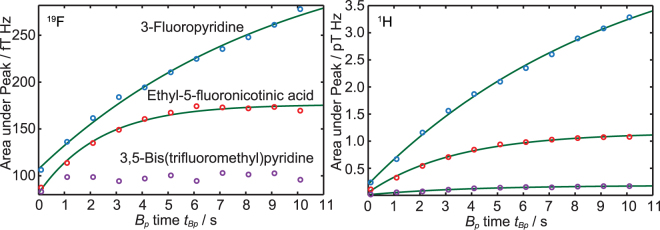
Integrated peak signals vs. pulse length *t*_*Bp*_ for the ^19^F (left) and ^1^H (right) MR signals of 3-fluoropyridine, ethyl-5-fluoronicotinic acid and 3,5-bis(trifluoromethyl)pyridine.

As a next characterization step, the signal enhancement as a function of the hyperpolarization time *t*_*Bp*_ was determined in order to gain the maximum SNR for multiple averages. The experimental setup was identical to the previous measurements; however, instead of sweeping *B*_*p*_, now *t*_*Bp*_ was varied between 100 ms and 10.1 s. The polarizing field *B*_*p*_ was fixed to the value where the maximum enhancement was observed (Fig. 4). The corresponding experimental parameters are listed in Table 1, rows 7 to 12.

An exponential saturation function

1 $$M({t}_{Bp})={M}_{HP}(1-\exp (-\frac{{t}_{Bp}-{t}_{0}}{{T}_{HP}}))$$

was fitted to the data (see green lines in Fig. 4). Here, *M*(*t*_*Bp*_) is the magnetization of the sample, which is proportional to the area under the peak. *M*_*HP*_ is the saturation magnetization for infinite polarizing time, *t*_0_ is a time offset and *T*_*HP*_ is the buildup time of the magnetization. The buildup time *T*_*HP*_ depends also on the longitudinal relaxation time *T*_1_, which is dependent on the *B*_*p*_ field strength, as well as on the buildup time constant for the hyperpolarization of the substrate. *T*_*HP*_ is dominated by the shorter of both processes. The fitted values for *T*_*HP*_ of the ^19^F and ^1^H signals of the three substrates are listed in Table 2.

**Table 2:** ^1^H and ^19^F ultra-low-field build up times for ethyl-5-fluoronicotinic acid, 3-fluoropyridine and 3,5-bis(trifluoromethyl)pyridine.

**Sample**	**Nucleus**	***T***_***HP***_ **/ s**
Ethyl-5-fluoronicotinic acid	^19^F	2.4 ± 0.5
3-Fluoropyridine	^19^F	9.4 ± 3.9
3,5-Bis(trifluoromethyl)pyridine	^19^F	—
Ethyl-5-fluoronicotinic acid	^1^H	3.4 ± 0.5
3-Fluoropyridine	^1^H	9.7 ± 2.4
3,5-Bis(trifluoromethyl)pyridine	^1^H	3.9 ± 1.0

The results show that *T*_*HP*_ can be different for the observed nuclei. Whereas for 3-fluoropyridine, *T*_*HP*_ for ^19^F and ^1^H is within the confidence interval and has in comparison to the other two substrates a very long *T*_*HP*_, the other two substances show a different behavior. *T*_*HP*_ for ^1^H of ethyl-5-fluoronicotinic acid is about 1 s shorter than for ^19^F, also within the confidence interval. The influence of the substituent (-COOCH_2_CH_3_) in ethyl-5-fluoronicotinic acid is even stronger and the *T*_*HP*_ time for ^19^F of 3,5-bis(trifluoromethyl)pyridine was too short and could not be fitted (see Fig. 4). As can be seen in Fig. 2 (purple dots) the hyperpolarization process for ^19^F is independent from the field strength. For the sequence shown in Fig. 1d the hyperpolarization process starts during the data acquisition time of the previous shot directly after the 90° pulse resulting in a minimum hyperpolarization time of the data acquisition time. For short *T*_*HP*_ the signal is already saturated. Therefore, a fit using Equation (1) will not lead to reasonable results. For the other substances, even though the hyperpolarization processes start also at the same position, they are not saturated for short *t*_*BP*_. Therefore, by varying *t*_*BP*_, Equation (1) leads to reasonable results.

**Figure 5:**
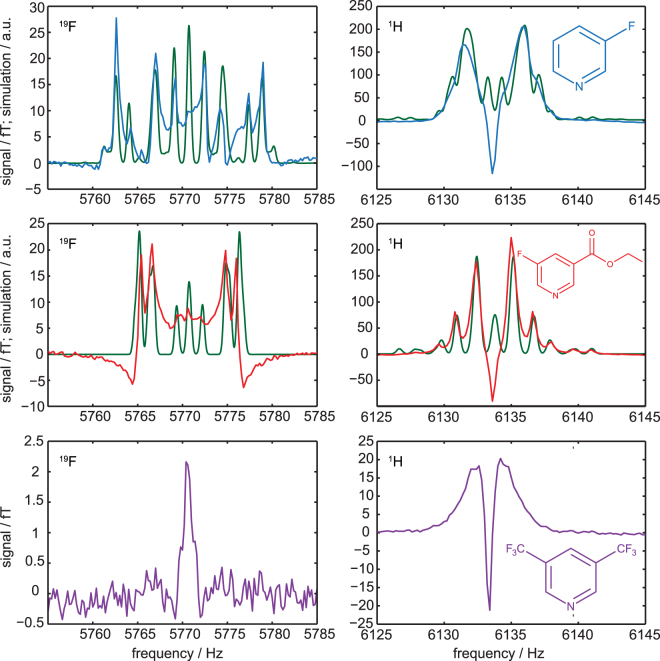
Ultra-low-field ^19^F and ^1^H MR spectra of hyperpolarized 3-fluoropyridine (upper row), ethyl-5-fluoronicotinic acid (middle row), and 3,5-bis(trifluoromethyl)pyridine (lower row). Substances and catalysts were dissolved in methanol and measured at 144 µT. Signals around 5770 Hz can be assigned to the ^19^F nuclei showing the ^1^H- ^19^F coupling. Signals around 6134 Hz can be assigned to the ^1^H signal. The blue, red and violet lines represent the measured spectra whereas the green lines represent simulated spectra based on high-field determined coupling constants.

In order to get more information on the hyperpolarization process, hyperpolarized high-resolution ULF spectra were additionally acquired using the simple FID sequence. The sequence parameters were set to the *B*_*p*_ amplitude for maximal enhancement, and *t*_*Bp*_ was roughly estimated to get the highest SNR for multiple averages. The sequence parameters are listed in Table 1 (rows 13 to 18). A detailed analysis of the ^1^H and ^19^F frequency regions in the ULF spectra show J-coupling resolved multiple resonances for the first two hyperpolarized substrates. For 3,5-bis(trifluoromethyl)pyridine only two singlets were observable (see Fig. 5).

To differentiate the hyperpolarization effects from the standard spectra, which were obtained at thermal equilibrium, simulations of NMR spectra without hyperpolarized signals were performed both at high and ultra-low fields using Bruker Topspin software (high-field) as well as VeSPA (low-field)^54^. The coupling constants were determined using thermal ^1^H and ^19^F spectra acquired at high-field (7 T) of all three non-hyperpolarized substrates dissolved in methanol-d_4_ (see supplementary material). Interestingly, the thermal simulations fit well even to the quite complex ULF hyperpolarized spectra (Fig. 5, green lines) except for a central resonance with large amplitude in the ^1^H spectrum. This line shows most likely hyperpolarized methanol. The ^19^F signal in the spectrum displays the ^1^H- ^19^F couplings, while ^1^H-^1^H as well as ^1^H- ^19^F couplings dominate the ^1^H spectrum. For 3,5-bis(trifluoromethyl)pyridine no J-couplings were detected. It may therefore be hypothesized that the effect of hyperpolarization significantly increases the signal at ULF but alters relatively little the overall spectral characteristics, except for the ratio of the amplitudes of several spectral lines. An analysis of the methanol resonance line in the ^1^H data supports the conclusion that the central line in the ^1^H spectral range is due to hyperpolarized methanol. This signal is also visible in the ^1^H ULF spectrum of 3,5-bis(trifluoromethyl)pyridine. However, as no J-couplings were detectable at high-field simulations, no simulations were performed for this substance. The broad ^1^H signal around the narrow central peak may be due to ^1^H resonances with shortened T_2_-time.

These encouraging results show that SABRE in combination with SQUID-based ULF NMR is well suited to increase the low SNR significantly and allow to measure otherwise non-detectable multinuclear spectra.

Aside from allowing measuring samples with low spin densities or nuclei with low sensitivity, the use of SABRE as a hyperpolarization technique enables a steady state generation of hyperpolarization. Compared to standard PHIP, SABRE allows repeating the experiment multiple times thus additionally increasing the SNR.

**Figure 6:**
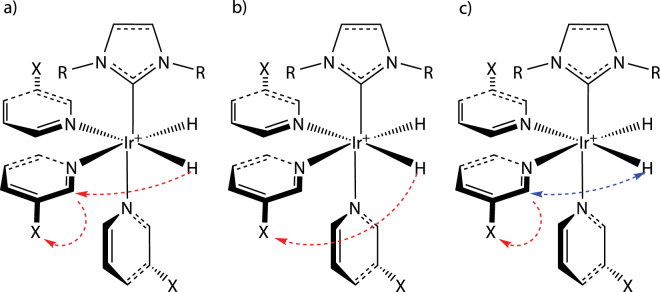
Possible polarization transfer mechanisms to spin-1/2 heteronuclei of pyridine derivatives. (**a**) Indirect polarization transfer: the polarization of hydrogen is transferred to hydrogen of the pyridine derivative and subsequently to the corresponding heteronucleus X, e.g ^19^F. (**b**) Direct polarization transfer: the polarization is transferred directly to the corresponding heteronucleus X of the exchangeable ligand. (**c**) Polarization transfer after H/H exchange reaction. Hydrogens of a pyridine ligand in ortho-position can exchange during SABRE reaction^56^ which would also enable a direct polarization transfer from polarized hydrogen atoms (to the corresponding heteronucleus of the exchangeable ligand X (here ^19^F).

Our measurements also demonstrate that the theory behind SABRE is not yet completely understood^11,22,23,52^. J-coupling as the main mechanism for the polarization transfer cannot explain the differences of the *B*_*p*_ field dependencies between the ^19^F and ^1^H signals of all three substrates. One would expect that the polarization is first transferred to a proton of the substrate and subsequently transferred to ^19^F (see Fig. 6a). But then the *B*_*p*_ dependencies of the ^19^F and ^1^H signal should be correlated, which is not the case. It seems that other coupling mechanisms such as dipole–dipole coupling cannot be neglected, since the polarization transfer takes place in the vicinity of the catalyst. Therefore, the secular part of the intramolecular dipole–dipole coupling does not average to zero, as it is the case in isotropic liquids^55^. Thus, a direct polarization transfer reaction from ^1^H to ^19^F might become possible (see Fig. 6b).

A further explanation of the observed results is based on a proton-proton exchange reaction (see Fig. 6c). It was reported that the ortho-standing hydrogen nuclei can exchange via the Ir-catalyst with Ir-coordinated hydrogen (here from parahydrogen)^56^. This supports the conclusion that at least one hydrogen atom from parahydrogen was transferred to the fluorinated pyridine derivative and followed by an intramolecular polarization transfer to the vicinal bounded fluorine. A complete simulation of the hyperpolarized spectra may therefore have to include additional processes.

The presented results demonstrate that a SQUID-based NMR system is a promising setup for the investigation of hyperpolarization techniques that work optimally in the μT and mT range and below, such as SABRE or dynamic nuclear polarization. As described by Appelt *et al*., low-field MR detection can be used for the determination of relaxation times, diffusion and J-coupling constants^57^. Our results also support the hypothesis of alternative reaction pathways in SABRE that are based on an H/H exchange^56,58^. Furthermore, SQUID-based systems can be used for magnetic resonance imaging and the simultaneous detection of different MR-active nuclei. Combining continuous hyperpolarization methods with SQUIDs increases the sensitivity to a point where spatially-resolved MR methods or even MR imaging of heteronuclei of background-free molecular probes at ultra-low-field conditions will be feasible.

The datasets generated and/or analyzed during the current study are available from the corresponding author on reasonable request.

## Electronic supplementary material

Supplementary Information
