# Supplementary material for: SQUID-based detection of ultra-low-field multinuclear NMR of substances hyperpolarized using signal amplification by reversible exchange
Source: Sci Rep. 2017 Oct 18;7:13431. doi: 10.1038/s41598-017-13757-7 (PMC5647402; doi:10.1038/s41598-017-13757-7)
Supplement: Supplementary file 1 — Supplementary Information [file 41598_2017_13757_MOESM1_ESM.doc]

SQUID-based detection of ultra-low field multinuclear NMR of substances hyperpolarized using signal amplification by reversible exchange

K. Buckenmaier^a^, M. Rudolph^a,b^, C. Back^b^, T. Misztal^c^, U. Bommerich^d^, P. Fehling^a^, D. Koelle^b^, R. Kleiner^b^, H. A. Mayer^c^, K. Scheffler^a^, J. Bernarding^d^ and M. Plaumann^d^

^a^ High-Field Magnetic Resonance Center, Max Planck Institute for Biological Cybernetics, Spemannstr. 41, 72076 Tübingen, Germany

^b^ Physikalisches Institut and Center for Quantum Science (CQ) in LISA^+^, University of Tübingen, Germany

^c^ Institute of Inorganic Chemistry, University of Tübingen, Germany

^d^ Department for Biometrics and Medical Informatics, Otto-von-Guericke-University, Magdeburg, Germany

**Overview**

1. Sample preparation

2. ^1^H-^19^F ultra-low field MR spectrum

3. ^1^H-^1^H and ^1^H-^19^F coupling constants of 3,5-bis(trifluoromethyl)pyridine, ethyl-5-fluoronicotinic acid and 3-fluoropyridine

**1. Sample preparation**

The three samples were prepared as listed in Table S1.

**Table S1: Composition of the sample solutions**.

| *Substrate:* | *3-Fluoropyridine** | *3,5-Bis(trifluoromethyl)pyridine*** | *Ethyl-5-fluoronicotinic acid** |
| --- | --- | --- | --- |
| *CAS number:* | 372-47-4 | 20857-47-0 | 22620-29-7 |
| *Molecular formula:* | C_5_H_4_FN | C_7_H_3_F_6_N | C_8_H_8_FNO_2_ |
| *M [g/mol]:* | 97.09 | 215.1 | 169.17 |
| *n [mol]:* | 0.00023 | 0.00023 | 0.00023 |
| *d [g/ml]:* | 1.13 |  | 1.197 |
| *V [ul]:* | 19.76 |  | 32.51 |
| *m [mg]:* | 22.3 | 49.47 | 38.91 |
|  |  |  |  |
| *[Ir(COD)(IMes)(Cl)] catalyst [mg]:* | 7.0 | 7.0 | 7.0 |
| *Methanol [ml]:* | 10 | 10 | 10 |

* Liquid mixed with methanol

** Powder dissolved in methanol

**2. ^1^H-^19^F ultra-low field MR spectrum**

Figure S1 shows an exemplary spectrum of ^1^H and ^19^F signals of hyperpolarized ethyl-5-fluoronicotinic acid. Due to the broad-band detection characteristics of the SQUID both nuclei can be detected simultaneously allowing a quantitative measurement of the magnetization as well as changes of the magnetization of both nuclei. For RF excitation a double-resonant pulse was used. TR of the sequence was 9.5 s with 50 averages. The *B_p_* field strength was 4.1 mT and *t_Bp_* was 4s.


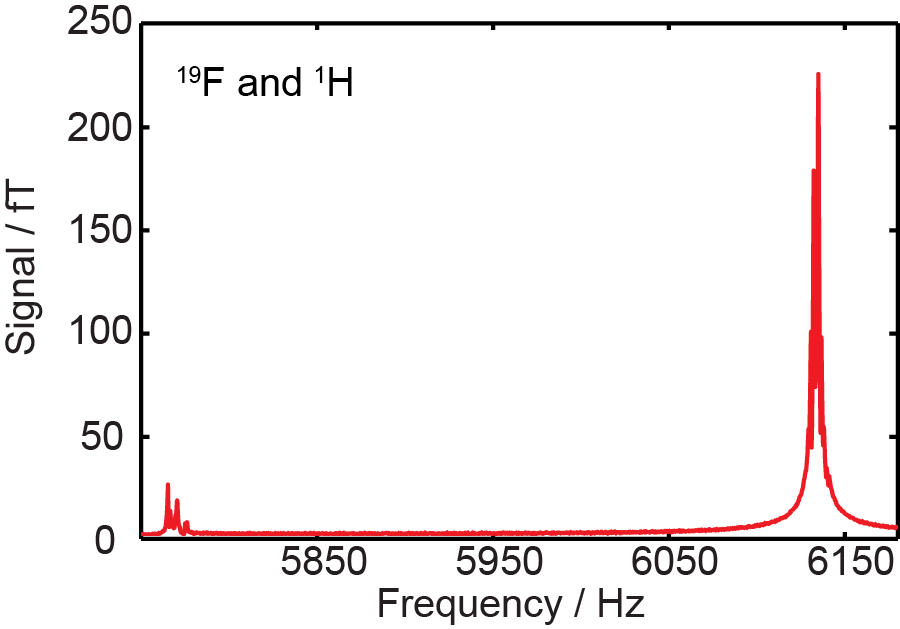


**Figure S1.** Simultaneous detection of hyperpolarized ^1^H (~6140 Hz) and ^19^F (~5770 Hz) nuclei of ethyl-5-fluoronicotinic acid under ultra-low field conditions using a SQUID-based detector. The absolute value of the MR signal is plotted (for high-resolution spectra cf. fig. 5 in main text middle panel).

**3. ^1^H-^1^H and** **^1^H-^19^F coupling constants of 3,5-bis(trifluoromethyl)pyridine, ethyl-5-fluoronicotinic acid and 3-fluoropyridine**

The ^1^H-^1^H or ^1^H-^19^F coupling constants were measured in a standard high-field NMR spectrometer with polarizing field of 7T (Bruker WB-300 system with AVANCE 3 unit, Bruker BioSpin GmbH, Rheinstetten). The substrates were mixed respectively dissolved in a 10mm NMR tube filled with 2ml methanol-d_4_. After adding the catalyst [Ir(COD)(IMes)(Cl)] the solutions were measured.

**^1^H-^1^H and ^1^H-^19^F coupling constants of 3,5-bis(trifluoromethyl)pyridine**

No ^1^H-^1^H or ^1^H-^19^F couplings could be detected for 3,5-bis(trifluoromethyl)pyridine at 7T. The corresponding high field NMR spectra with signal assignment are shown in Figure S2.


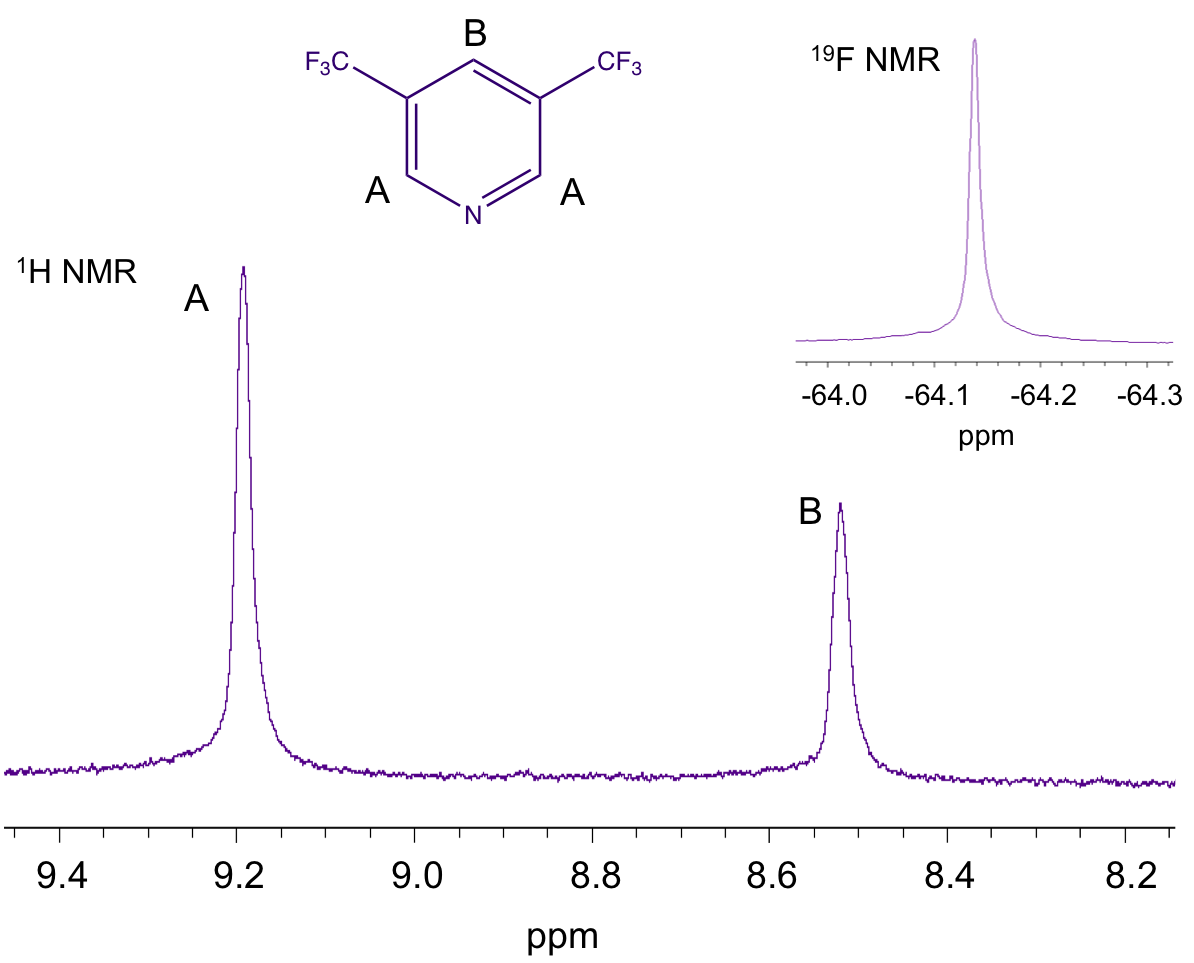


**Figure S2.** ^1^H and ^19^F MR spectra of 3,5-bis(trifluoromethyl)pyridine measured in methanol-d_4_ at 7T.

**^1^H-^19^F coupling constants of ethyl-5-fluoronicotinic acid**

Ethyl-5-fluoronicotinic acid was measured under the same conditions as 3,5-bis(trifluoromethyl)pyridine. The high-field ^1^H and ^19^F NMR spectra (7T) and determined coupling constants of ethyl-5-fluoronicotinic acid are shown in Figure S3.


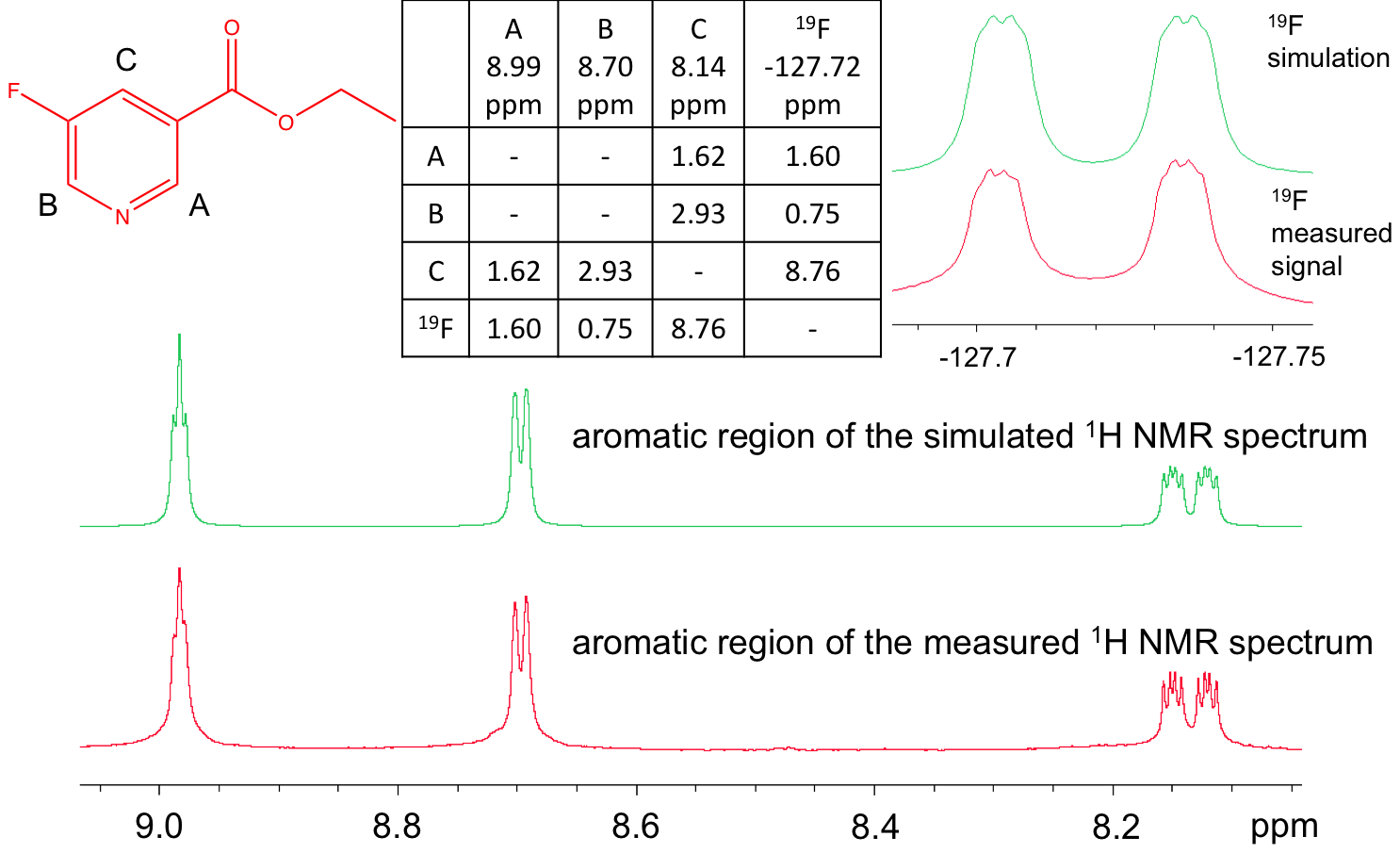


**Figure S3.** ^1^H and ^19^F MR spectra of ethyl-5-fluoronicotinic acid (red: measured in methanol-d_4_ at 7T; green: simulated spectra) and the determined corresponding coupling constants.

**^1^H-^19^F coupling constants of 3-fluoropyridine**

Figure S4 displays the ^1^H and ^19^F NMR signals as well as the simulated signals based on the coupling constants listed in the table.

**
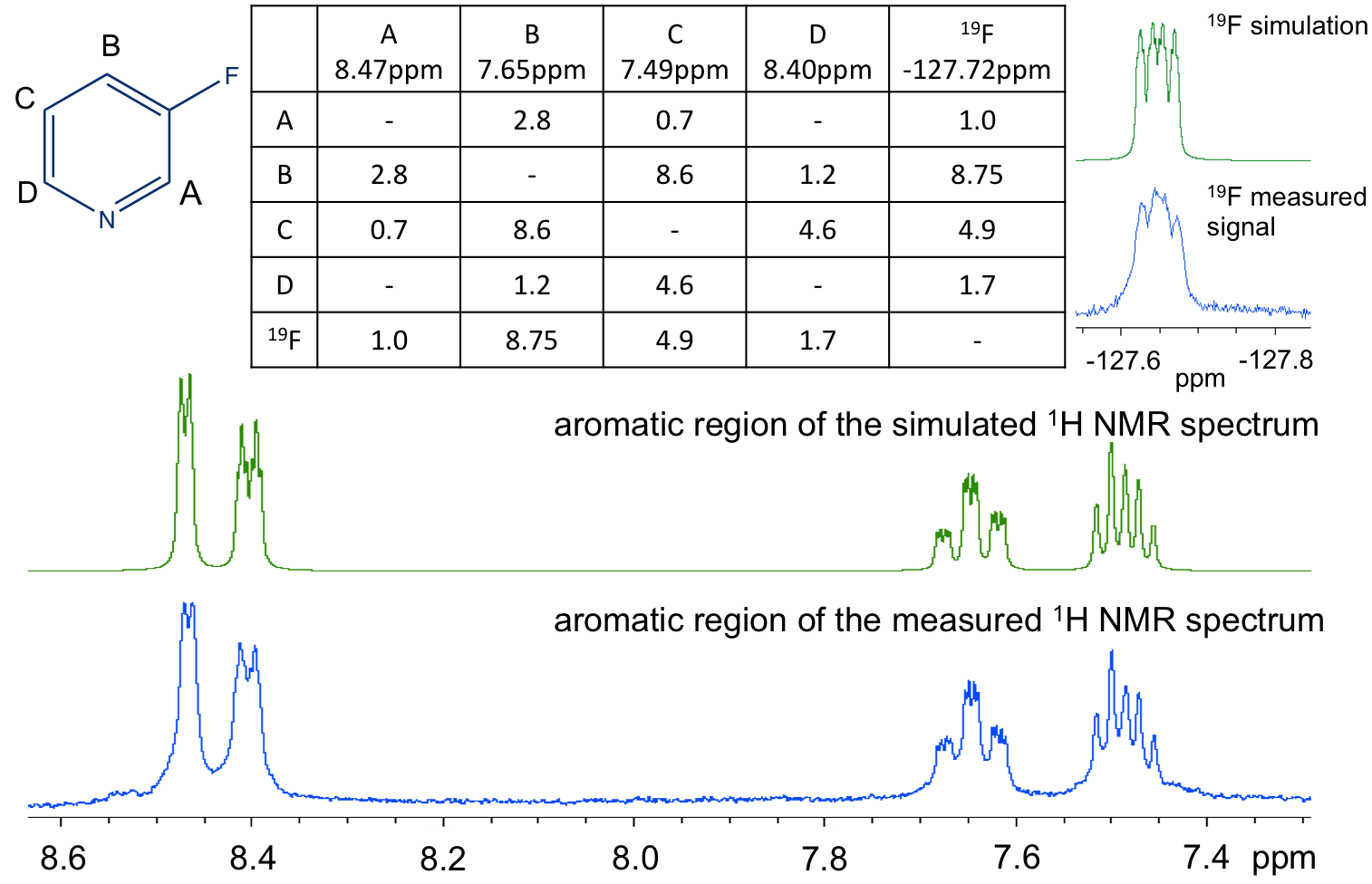
**

**Figure S4.** ^1^H and ^19^F MR spectra of 3-fluoropyridine (blue: measured in methanol-d_4_ at 7T; green: simulated spectra) and the determined corresponding coupling constants.
